# Experiences of COVID-19 dedicated ward nurse managers in South Korea: a qualitative study

**DOI:** 10.3389/fpubh.2025.1675436

**Published:** 2025-10-09

**Authors:** Rosa Yoon, Minkyung Gu, Mira Park, Sohyune Sok

**Affiliations:** ^1^Department of Nursing, Graduate School, Kyung Hee University, Seoul, Republic of Korea; ^2^Department of Nursing, College of Health Science, Daejin University, Pocheon-si, Gyeonggi-do, Republic of Korea; ^3^Department of Nursing, Graduate School, Kyung Hee University, Seoul, Republic of Korea; ^4^College of Nursing Science, Kyung Hee University, Seoul, Republic of Korea

**Keywords:** COVID-19, nurse managers, focus group, infection disease, qualitative approach

## Abstract

**Background:**

The outbreak of new infectious diseases is on the rise worldwide. In particular, hospital nurses and nursing managers responding to the COVID-19 pandemic are facing overwhelming workloads amidst confusion and anxiety. In South Korea, qualitative research exploring the experiences of nurse managers in COVID-19 dedicated wards are needed to strengthen their capacity and develop response strategies.

**Objective:**

This study aimed to comprehensively understand and explore the experiences of nurse managers in COVID-19 wards.

**Methods:**

A qualitative study using the phenomenological research method of Giorgi was employed. Participants were 12 nurse managers who had been performing nursing care in a dedicated ward for the treatment of COVID-19 patients for more than 1 month and had at least 2 years of experience as a COVID-19 nursing unit manager at general hospitals. They were randomly assigned to two groups of six participants for focus group interviews, and they were in-depth interviewed until data saturation. Data were collected from April to July, 2022.

**Results:**

The duration of experience as nurse managers in COVID-19 dedicated wards was an average of 7 years and 5 months. The duration of experience as staff nurses in COVID-19 wards was an average of 1 year and 2 months. The five themes derived from this study were ‘the opening of unprecedented COVID-19 dedicated wards’, ‘difficulties in personnel management’, ‘overall predicaments as managers’, ‘demonstrating leadership with responsibility’, and ‘pride in overcoming hardships’. Also, eleven sub-themes were derived.

**Conclusion:**

Nurse managers in COVID-19 dedicated wards need to establish rules and guidelines within the ward to prevent and reduce confusion. They also need to collaborate with hospital administrators to ensure that staff nurses assigned to COVID-19 dedicated wards receive relevant training and are assigned accordingly. Intervention programs for COVID-19 dedicated ward nurse managers need to be developed and implemented. Policymakers need to develop government-level policies and systems to establish a medical support system for hospitals operating COVID-19 dedicated wards.

## Introduction

In December 2019, a novel infectious disease called COVID-19 (Coronavirus Disease-2019) was first reported in Hubei Province, China, and it rapidly spread worldwide (1). As a result of this unexpected outbreak, an unprecedented COVID-19 infection-focused healthcare system was put into place. Administrative orders were issued to secure hospital beds for the treatment of multiple COVID-19 patients, mainly in general hospitals and national/public hospitals. Of note, nurses make up 59% of healthcare workers, and they were essential in providing COVID-19 patients with nursing care (2, 3). They also carried out important public health responsibilities, such as preventing and managing COVID-19 infections, and providing care for people in self-quarantine and confirmed patients. However, this dangerous situation could even lead to the collapse of the medical system in an unusual way, especially with the spread of COVID-19 infections among nurses and healthcare professionals (4). Due to the shortage of nursing staff, no significant progress was made in the frontline healthcare settings beyond the commitment and selflessness of nurses. The stress associated with COVID-19 led to emotional and health problems, and burnout among nurses, physicians, and other healthcare workers, thus creating an excessive load for healthcare workers (5, 6).

Meanwhile, COVID-19 dedicated ward nurse managers were occupied with revising and developing infection control and prevention guidelines in order to protect the nursing staff specializing in COVID-19 during the widespread outbreak, in addition to fulfilling their previously assigned roles as nursing administrators and practitioners. First, they endured significant stress and challenges while addressing various difficulties in training and managing nursing personnel to enhance COVID-19 infection education and job competency in the COVID-19 dedicated ward (7, 8). The nurse manager also needed to consider staff reassignment as part of ward operations to ensure that the nursing staff were appropriately suited for the COVID-19 dedicated ward. Deploying skilled nursing staff to care for infectious COVID-19 patients was a challenging decision for the nurse manager, who held significant responsibilities (9, 10). However, despite the importance of the frontline nurse manager’s role in the COVID-19 situation, there is no available information on the direct struggles of COVID-19 dedicated ward nurse managers.

Most of the studies published to date regarding COVID-19 have focused on the experiences of general nurses as they provided patient care amid excessive workloads and infection-related anxieties (3, 4, 9, 11–13). Several studies on nursing staff during COVID-19 indicate that the actual COVID-19 responses of hospitals are relatively distant from the organizational management related to the administration of COVID-19 dedicated ward nurse managers, especially in areas such as bed operation, personnel management, and support of control tower operations (14–17). On the other hand, COVID-19 dedicated ward nurse managers were responsible for coordinating nursing care amid the uncertainties of COVID-19 and frequent changes in guidelines, as they played a central role in infection management system. They also had to perform numerous nursing tasks with clearly defined roles and responsibilities, or to continuously take on new roles without any training or education related to COVID-19 (8, 18). Furthermore, they struggled to conduct safety training for the nursing staff who lacked expertise and to manage a grievance system for the hospital administrative staff and patient guardians in an integrated manner (7, 8). One of the main concerns of COVID-19 ward nurse managers was the complete lack of information about COVID-19. Nurse managers had to rely on limited human and material resources in order to safeguard their lives and those of the nursing staff because of the lack of confidence in COVID-19 safety management (2, 19). Therefore, it is crucial to explore the experiences of COVID-19 ward nurse managers in a practical and impactful manner. This study aims to assist in providing clear responses based on the roles of managers and to facilitate prompt nursing care for infected patients in the event of new infectious disease situations that may arise in the future. Additionally, we seek to effectively operate a customized medical support management system in collaboration with the nursing staff so as to optimize the use of financial resources in a safe nursing environment and to take the lead in nursing care quality. This study is also conducted in order to help establish a comprehensive nursing administrative strategy for nurse managers, thus allowing nurses at the forefront of nursing organizations to focus solely on their duties.

The purpose of this study was to comprehensively explore the experiences of nurse managers in COVID-19 wards to establish nursing administration strategies and operate an effective medical support management system in preparation for future outbreaks of new infectious diseases. The research question was, “What is your experience in ward management as a dedicated ward nurse manager in the COVID-19 situation?”

## Methods

### Study population

A qualitative study using the phenomenological research method of Giorgi (20) was employed, and adheres to the Consolidated Criteria for Reporting Qualitative Studies (CORE-Q) (See Supplementary File 1). A qualitative approach was selected for understanding and exploring the experiences of nurse managers working in COVID-19 dedicated wards from their perspectives and analyze in-depth meanings. The participants in this study were selected based on their nursing care experience in dedicated wards for COVID-19 patients for more than 1 month following the government’s order to secure dedicated treatment beds for severe COVID-19 patients. They also had at least 2 years of experience as nursing unit managers for COVID-19. The selection of participants was conducted through a convenience sampling method, where the purpose and intent of the study were explained to the heads of the nursing departments at general hospitals and national/public hospitals in Seoul and Gyeonggi-do. The nursing department heads were then asked to recommend nurse managers who could eloquently and fully articulate their experiences in managing COVID-19 dedicated wards. In order to encompass diverse experiences related to the management of COVID-19 dedicated wards, the number of study participants was based on prior research by Krueger and Casey (20), which suggested a suitable number of five to eight participants per focus group interview. Meanwhile, 12 COVID-19 dedicated ward nurse managers were recruited and randomly assigned to two groups of six participants for focus group interviews in order to ensure this study’s integrity.

### Data collection

In this study, the interview questions were primarily prepared to gather comprehensive information from COVID-19 dedicated ward nurse managers in each hospital (See the Appendix). The interview questions were accompanied by additional questions concerning various situations, problem-solving, and cases related to the COVID-19 dedicated ward nurse managers. Three times were conducted for each group, resulting in a total of six focus group interviews. Each in-depth interview was conducted by one of the researchers. The once interviews lasted from 80 to 120 min, with an average of approximately 100 min, and in-depth interviews were conducted until data saturation was reached. The interviews were conducted via non-face-to-face Zoom video conferencing based on the national COVID-19 prevention situation. These sessions were mainly held in locations convenient for the participants, such as their offices or homes. The participants were then notified of the interview schedule and sent the accessible link via email. On the day of the interview, each participant’s attendance was reconfirmed in order to ensure that everyone was connected online before proceeding. First, the study began with an introduction of the researchers, followed by a comprehensive explanation of the research purpose and the interview process. The content of the interview was recorded with voluntary consent. In order to encourage the participants to freely share their experiences, the interview started with an open-ended question such as, “Could you please describe your experience in managing the dedicated ward during the COVID-19 situation?” (18, 20). The interview commenced with casual conversations, such as self-introductions. Once familiarity was established, we connected the flow of material by using open and unstructured questions. Furthermore, intentional questions were excluded in order to maintain an open attitude and objectivity and to avoid the researcher’s biases. In the final stage of the interview, all key contents were shared with the study participants so as to confirm the accuracy of the information conveyed. If further clarification was deemed necessary, we obtained the participants’ consent and conducted follow-up phone interviews. The assistant researcher transcribed the interviews on the day of the meeting and read the transcribed content repeatedly in order to enhance the accuracy of the data. In this study, data saturation was confirmed after conducting focus group interviews with 12 participants, and all processes related to data collection were concluded. Data were collected from April to July, 2022.

### Data analysis

The data analysis of this study was conducted by using the inductive analysis method among the qualitative research methods so as to enhance a broad understanding of the phenomenon. The transcribed interview data was processed through the following procedures. The data collected from the study participants were repeatedly read to grasp the overall meaning, and ambiguous parts of the statements were clarified through re-questioning to specify their meaning. Additionally, words, phrases, and sentences were extracted from meaningful statements to generate codes. The relevance and differences of the codes generated within the statements were analyzed, while related codes with similar meanings were grouped in order to create subtopics. Core content and concepts that could represent the classified subtopics were derived, and different topics were completed by using concise abstract words or phrases. They were ultimately grouped in order to complete themes. The completed themes provided a comprehensive description of the general research phenomenon based on the research content. All researchers participated in the data analysis process. When disagreements arose among researchers, they reconfirmed the essential meaning of the data with the interviewees, shared their findings, and then agreed on a consensus through the analysis process.

### Ensuring the validity of the study

In order to secure the integrity of the study, the four elements, i.e., reliability, transferability, dependability, and confirmability, were used in accordance with the validity evaluation criteria for qualitative research proposed by Lincoln and Guba (21). (1) Reliability pertains to the confidence in the truthfulness of the study findings. This means ensuring that the researcher accurately reflected the participants’ perspectives in the results. In this study, the researcher interviewed nurse managers who could speak about their management experience after directly overseeing COVID-19 wards. In order to ensure reliability, the participants reviewed and clarified cases where the meaning of the transcribed material was unclear to them. During the data analysis process, patterns were analyzed, including any contradictory data, and codes that potentially did not fit the established themes were classified and verified as “others.” (2) Transferability indicates the potential applicability of findings in different contexts, demonstrating a thorough description of the phenomena. In this study, the researcher used exploratory questions during interviews in order to obtain detailed statements regarding not only the participants’ surface behaviors, but also the contextual meanings of those behaviors. (3) Dependability signifies that results can be consistent and repeatable. In this study, the researcher constantly discussed the study process, analysis, and results with one nursing professor who has over 10 years of qualitative research experience, and collaborated on the data analysis process. (4) Confirmability refers to the degree of neutrality, or the extent to which the findings are influenced by the respondents rather than by the researcher’s biases, motives, or interests. In order to maintain neutrality, the researcher systematically documented decisions pertaining to the research methodology, implementation plans, and biases in a research journal. A variety of data were also utilized for analysis and interpretation so as to identify blind spots in the analysis and to enhance the understanding of the diversity of data interpretation.

### Ethical considerations

This study was conducted after going through the review and approval process of Kyung Hee University Institutional Review Board (IRB No. KHSIRB-22-055(RA), Approval date: April 4, 2022) to ensure the ethics of the participants. Before starting the interview, the researcher explained to the participants the purpose and method of the study, the participants’ rights, and the Zoom video recording and audio recording of the interview in accordance with research ethics. In addition, it was explained that the collected data would not be used for purposes other than the study, and that withdrawal from the study was possible at any time during the study without any disadvantages resulting from this. It was informed that the Zoom video files, audio files, and transcripts of the interview would be kept for 3 years from the end of the study, after which the paper documents would be destroyed using a document shredder, and the electronic documents would also be permanently destroyed.

## Results

### General characteristics of the study participants

This study involved 12 nurse managers with experience working in general hospitals and public hospitals that had newly established dedicated wards for the treatment of severely ill COVID-19 patients. The ages of the 12 nurse managers ranged from 38 to 58 years old, with an average age of 51.4 years, and all were female. The education levels of the study participants included 1 with a bachelor’s degree and 11 with a master’s degree or higher. The duration of clinical experience as nurse managers in COVID-19 dedicated wards ranged from a minimum of 2 years and 2 months to a maximum of 18 years, with an average of 7 years and 5 months. The duration of clinical experience as staff nurses in COVID-19 dedicated wards ranged from a minimum of 2 months to a maximum of 2 years and 1 month, with an average of 1 year and 2 months (Table 1).

**Table 1:** General characteristics of the study participants.

No	Gender	Age (year)	Level of education	The duration of clinical experience as nurse managers in COVID-19 dedicated wards (year/month)	The duration of clinical experience as staff nurses in COVID-19 dedicated wards (year/month)
1	Female	54	Bachelor’s	7/1	2/1
2	Female	38	Master’s	2/2	0/2
3	Female	41	Master’s	4/1	1/7
4	Female	52	Master’s	4/1	0/3
5	Female	52	Master’s	16/1	0/4
6	Female	53	Master’s	11/0	2/1
7	Female	54	Master’s	7/1	2/1
8	Female	54	Master’s	10/2	0/2
9	Female	54	Master’s	3/9	1/4
10	Female	54	Master’s	3/0	1/5
11	Female	56	Master’s	8/0	0/2
12	Female	58	Master’s	18/0	2/1

### Situational structural description of study participants

The analysis of focus group interviews exploring the management experiences of COVID-19 dedicated ward nurse managers yielded 240 meaningful statements. From these statements, 36 codes were formed. These codes were subsequently grouped into five themes and 11 subthemes as they were organized into more abstract and comprehensive groups of similar meaning. The detailed content for each theme and its corresponding subthemes are as follows (Table 2).

**Table 2:** Experiences of COVID-19 dedicated ward nurse managers.

Theme	Subthemes	Formulated meaning
1. The opening of unprecedented COVID-19 dedicated wards	1. Pressure of creating something out of nothing 2. Confusion due to frequent changes	Transfer to a different department without any mental preparation The current department is being converted to a COVID-19 dedicated ward Creating a new environment in a short period of time without a model ward Daily changes without clear standards for infection control policies and guidelines
2. Difficulties in personnel management	1. Difficulty in managing personnel deployed without proper planning 2. Difficulty in operating without complete staffing	The difficulty of managing personnel regardless of career experience A schedule that needs to be made dozens of times Too many nursing staff to manage alone
3. Overall predicaments as managers	1. Consumed by endless tension 2. Helplessness of being out of control 3. Managers who are misunderstood	Exhausted from excessive work Feeling limited by situations beyond your control Struggling with nurses’ incomprehensible demands The stress of having to mediate numerous conflicts
4. Demonstrating leadership with Responsibility	1. Communicating for effective management 2. Implementation of training for managing safe nursing environments	Sharing information in various ways to ensure department stability Actively communicate to resolve conflicts Operating programs to promote safe work sites
5. Pride in overcoming hardships	1. Companionship found amid the tsunami 2. Experiencing gem-like potential during chaos	Demonstrate strength with the support of department members Gain confidence and not be afraid of difficulties Discovering the hidden potential of your subordinates

### The opening of unprecedented COVID-19 dedicated wards

The dedicated ward that was operated under administrative orders during the COVID-19 situation was determined to be a hospital with established medical personnel and various logistical facilities and environments. Moreover, negative pressure isolation facilities were constructed for patients who were transferred to places without treatment facilities, such as general wards, rather than intensive care units. The nurse managers were assigned to dedicated wards without any mental preparation, facing the difficulty of having to prepare and operate within a short timeframe and without clear standards or guidelines related to the opening of the COVID-19 dedicated ward. Furthermore, nurse managers experienced considerable confusion when confronted with new tasks, such as developing work process manuals for COVID-19 hospitalized patients and establishing relevant guidelines.

#### Pressure of creating something out of nothing

*“One day, I suddenly said, ‘Starting tomorrow, I should go there and start working,’ and I had been preparing since February to open the COVID-19 ward in March… then, I arrived suddenly. (excerpt) The first year was really, really hard… it felt like I was a test subject.”*

#### Confusion due to frequent changes

*“According to administrative orders, hospital guidelines really change frequently, even in the morning and evening. First of all, there is no established manual or standard, so…”*

The first category in the subthemes, ultimately confirmed through the above statement, is as follows: The unexpected opening of a dedicated COVID-19 ward left nursing managers feeling confused and anxious. They faced challenges as nursing managers due to the lack of clear guidelines and the flawed guidelines, which led to a chaotic and chaotic environment.

### Difficulties in personnel management

The hospital’s own nursing staff was assigned to run the site; however, an unparalleled period of pandemonium ensued as verified cases overlapped with nursing workers. The nurse managers faced significant challenges in managing an unexpected influx of more than 100 nurses sent from various locations. The COVID-19 dedicated ward was experiencing problems while the head nurse frequently took over responsibilities, adjusted staffing, and repeatedly revised the work schedule.

#### Difficulty in managing personnel deployed without proper planning

*“Since the nurses transferred over 100 people from one ward to another, serious territorial issues emerged, and because they have different experiences, it was challenging to combine them. As we were unfamiliar with one another while working together, we frequently found ourselves in conflicting situations.”*

#### Difficulty in operating without complete staffing

*“At first, we managed the COVID-19 dedicated ward. Once the situation improved, we assumed the roles of general ward managers. However, when the number of COVID-19 patients increased again, we closed the general ward, and went back and forth… (excerpt)”*

The second category in the subthemes, ultimately confirmed through the above statement, is that nursing staff dispatched from various locations were faced with unfamiliar tasks without clear procedures or systems. Furthermore, the lack of structure in their work and roles led to confusion when performing nursing duties. In other words, the final subtheme identified was the so-called conflict situation, which perpetuated a vicious cycle of unreasonable demands on human resources.

### Overall predicaments as managers

While preparing to open the COVID-19 dedicated ward, the nurse managers were mainly in charge of the basic preparations for the ward and personnel. Furthermore, they conducted training, provided supplies, and managed the facility almost by themselves. However, in prioritizing staff safety against infections during COVID-19, the nurse managers were compelled to enforce limitations on private gatherings and even regulate the employees’ personal lives, which proved to be extremely frustrating for them as nurse managers.

#### Consumed by endless tension

*“In fact, I have not thought about resigning until now, but while working as a manager in the COVID-19 dedicated ward, I thought it would really be hard to endure month after month. It was really not easy, and now that I think about how I spent each day, it is hard to recall—it was all a haze.”*

#### Helplessness of being out of control

*“It seems as though our nurses are being shot on the battlefield, falling, and contracting COVID-19, while the hospital provides minimal support. How are we expected to carry on in this scenario where we are forced to rely on the precarious lives of our nurses?”*

#### Managers who are misunderstood

*“Although I have extensive experience in clinical practice, I have never encountered a situation as challenging as this one. Several shocking events have taken place. How do you intend to address these concerns? How will you directly support us? Hmm… The daily allowance is not enough. At that time, I felt like there was nothing I could do.”*

The third category in the subthemes identified through the above statements is as follows. Nurse managers mentioned that, with the addition of new COVID-19 tasks to their existing duties, they felt helpless, unable to make decisions or take action, in a situation where they were at a loss. Furthermore, nurses who performed their duties expressed frustration at being dismissed as mere desk jockeys, ignorant of the practicalities of the situation, when seeking cooperation.

### Demonstrating leadership with responsibility

The nurse manager directly communicated verbally during handover time and also created a bulletin board to share requests, ensuring that communication was accurate and fair. They actively facilitated effective management by holding regular consultations, organizing meetings, and mediating conflicts among colleagues. Additionally, they prioritized the safety of employees by creating a communication platform focused on safety management to work towards specific solutions.

To ensure accurate and fair communication, the nurse managers either created a bulletin board to convey requests or spoke directly with the staff during handover time. They were actively communicating for efficient management by setting up meetings, conducting frequent consultations, and arbitrating disputes among coworkers. They also prioritized employee safety by establishing a forum for safety management discussions aimed at developing targeted solutions.

#### Communicating for effective management

*“I communicated openly while sharing continuously about what I could offer, what I could do, and what I needed, so it was still okay.”*

#### Implementation of training for managing safe nursing environments

*“I advised them to focus on ward administration while exercising caution to prevent accidents, particularly those affecting the safety of the employees and patients. I asked them to use the study room for training related to these topics.”*

The fourth category in the subthemes, ultimately confirmed through the above statement, was the recognition that, as a nursing manager, it was important to understand the difficulties faced by team members and to take responsibility for them to the end. The nursing manager worked to prevent further spread of infection through prompt identification and reporting, and built close relationships with department members to ensure a thorough understanding of the situation.

### Pride in overcoming hardships

The nurse managers reported increased pride and confidence in their experience as discussions focused on the COVID-19 pandemic and ward reduction plans. They would be capable of managing any new infectious disease that may arise in the future without worry.

#### Companionship found amid the tsunami

*“The strength I gained from working alongside the COVID-19 dedicated ward nurse manager has undoubtedly been a source of motivation for me. I believe what mattered most was the continuous support and companionship of my manager.”*

#### Experiencing gem-like potential during chaos

*“I now have a certain confidence that, despite any challenges, I can effectively manage nurses. Considering how frequently these kinds of incidents occur, it appears that this has given us a chance to grow considerably with our nurses.”*

The final fifth category in the subthemes, confirmed through the above statements, emphasized the importance of teamwork among nurse managers even during the COVID-19 crisis. The subtheme ultimately derived from this statement was that nurse managers realized the value of their calling as medical professionals through camaraderie and gained the confidence and pride that they could overcome any difficult situation.

## Discussion

In this study, the first theme was “The opening of unprecedented COVID-19 dedicated wards.” This supports a previous study indicating that the constantly changing nursing goals during the unpredictable COVID-19 infectious disease situation created confusion in operations management (8, 18, 22, 23). The nurse managers encountered situations where they were reassigned to different departments without mental preparation. They had to create manuals for severe COVID-19 patients despite the absence of clear standards or guidelines regarding COVID-19. Additionally, the nurse managers faced considerable confusion as they had to establish guidelines based on the incoming COVID-19 patients. In this regard, both nurse managers and nurses need to acquire a broad range of medical knowledge related to new infectious diseases. Moving forward, they must actively adhere to standardized guidelines while maintain composure, even in situations involving emerging infectious diseases (24). In particular, nurse managers should take the lead in adhering to new medical knowledge related to novel infectious diseases and ensure that the nurses can promptly fulfill their responsibilities (25). Furthermore, the nursing headquarters needs to view the difficulties faced by nursing managers as mediators between middle managers and general nurses with greater understanding and empathy. As Mansour and Shosha (26) noted in their study that the nursing managers’ ability to cope with crisis situations and their maturity are paramount, the nursing headquarters needs to grant and delegate greater autonomy to nursing managers so that they can solve work-related problems in a way that suits the characteristics of each nursing unit, beyond simply relaying directives from superiors and quelling resistance from general nurses.

The second theme was “Difficulties in personnel management.” One of the important aspects at the organizational level in responding to infectious diseases is securing an adequate nursing workforce. The global demand for nursing staff has increased as a result of COVID-19. This aligns with other research indicating that, without proper organizational support, ongoing individual-level attempts to respond to infectious diseases will eventually lead to burnout (11, 12, 26). Accordingly, in order to prevent burnout caused by work-related stress and to provide ongoing management that can promote mental stability, the nursing staff management must enhance the nursing work environment (5, 13). In particular, the conservative and hierarchical nursing culture must be improved to promote smooth communication between superiors and subordinates within the nursing organization and to improve unfair practices. Similar to the study by Chung et al. (15), which emphasized the paramount importance of the nurse manager’s interest and trust, nurse managers should strive to foster mutual respect to restore the self-esteem that may be diminished and lowered in new nurses, and take the lead in fostering a culture that boosts morale among nursing organization members based on trust.

The third theme was “Overall predicament as managers.” During the COVID-19 pandemic, dedicated ward nurse managers relentlessly exhausted their morale, as well as their physical and mental health, both professionally and personally. They were enmeshed in negative feelings and had to bear heavy duties without reservation. This finding aligns with other research showing that, when faced with overwhelming workloads and inadequate support networks, the study participants had to struggle alone in order to overcome their constraints (27). In the future, relevant departments and institutions need to pay more attention to the psychological and social needs of nurse managers by developing and implementing healing programs, such as meditation and relaxation therapy, to reduce their fatigue. Moreover, as mentioned in the study by Lee and Lee (14), it is assumed that nurse managers are also human beings who cannot be perfect, and nurses themselves need to find specific strategies to change their thinking toward nurse managers in a positive way and find easier causes and methods to deal with managers.

The fourth theme was “Demonstrating leadership with responsibility.” The nurse managers have shown their ability to foster leadership by enabling the nursing staff to focus on their core responsibilities during COVID-19. This is consistent with the study conducted by Lee and Lee (14). They noted that the nurse managers experienced a high level of physical and mental stress while responding to COVID-19; however, they were able to validate their potential through a strong sense of responsibility and accomplishment. Hence, personality enhancement programs that promote health through post-traumatic growth and development and help people realize the importance of nursing should be implemented and actively promoted for greater use (15). In particular, it is more important to create an environment for nursing work that supports mutual respect and consideration (12), and it is necessary to operate periodic programs that can enhance the self-efficacy of nursing managers themselves, so that they can demonstrate leadership with a positive mindset.

Lastly, the fifth theme was “Pride in overcoming hardships.” The nurse managers in this study identified several initial challenges they encountered. However, they also shared invaluable experiences of adapting despite the difficulties, which renewed their hope of overcoming any future crises caused by novel infectious diseases. This corroborates studies showing that trust and respect among coworkers facilitate overcoming challenges, even when faced with COVID-19 (15, 16). In conclusion, this study highlights the importance of nurse managers on each ward for nurses who are caring for COVID-19 patients, and the need for strong support networks that address the diverse psychological requirements of nurses. To ensure that the nurses receive adequate financial support within a safe working environment, the nurse managers must recognize the stress that the nurses experience due to the intense pressures associated with COVID-19 (17, 28, 29). In other words, it is necessary to establish an efficient and productive nursing unit work system, and furthermore, to enhance empathy to communicate with the new generation of young nurses. Nurse managers must strive to develop their capabilities to cultivate qualities that combine profound personal qualities.

### Implications for practice, policy, and research

The results of this study can be used as basic data for establishing a safe medical support system for nurse managers in response to new infectious diseases in the future and for resource management strategies according to nursing personnel. In addition, it can be used to widely publicize the necessity of infectious disease response strategies in preparation for the outbreak of new infectious diseases and to develop policies that can improve the quality of nursing for infected patients. Based on the results of this study, we suggest a follow-up study that can develop a program to reduce post-traumatic stress from the work of nurses by department that reflects the characteristics of Korean hospitals by expanding the experience of nurse managers in COVID-19 wards and validly verifying it.

## Limitations

There were limitations to this study. The sample was quite small (12 participants) and limited to a specific region. This study was conducted via non-face-to-face video interviews due to compliance with the government’s quarantine guidelines and the infection control guidelines of medical institutions. Therefore, the interviews may have been directly or indirectly affected by the status of personal computers and other devices. Because the in-depth interviews were conducted remotely, it was difficult to accurately capture nonverbal expressions, which could have influenced the research results. Furthermore, the interaction and empathy between the interviewer and the participants were limited due to the remote nature of the interview. In addition, it may have been difficult for the participants to honestly express their opinions or feelings, considering the position of the hospital where they currently work as managers of the nursing organization. The researcher encouraged the participants to freely share their experiences, but there may be limitations due to restrictions on expression. These factors may be limitations of this study.

## Conclusion

Based on the above research results, nurse managers need to establish rules and guidelines within the ward to prevent and reduce confusion and ensure that decisions regarding COVID-19 dedicated wards are not constantly changed. They should also collaborate with hospital administrators in personnel management to ensure that staff nurses assigned to COVID-19 dedicated wards receive relevant training. Intervention programs should be developed and implemented to strengthen infectious disease response capabilities, human resource management, empowerment, communication skills, and leadership for nurse managers in COVID-19 dedicated ward. Policymakers need to develop government-level policies and systems to establish a healthcare support system for hospitals operating COVID-19 dedicated wards, including safe environment management, human resource management, dedicated ward management by nurse managers, strengthening nurse competency, and teamwork and cooperation among relevant medical and welfare teams. In a situation where most of the previous studies related to clinical nurses in COVID19, this study is significant and valuable in that it attempted qualitative research targeting nursing managers in a COVID19 dedicated ward.

## Data Availability

The original contributions presented in the study are included in the article/Supplementary material, further inquiries can be directed to the corresponding author.

## References

[1] World Health Organization. WHO press conference on COVID-19 and other global health issues. World Health Organization, Geneva, (2023). Available online at: https://www.who.int/multi-media/details/who-press-conference-on-covid-19-and-other-global-health-issues---5-may-2023 (accessed May 28, 2024)

[2] Deldar K Froutan R Ebadi A. Nurse managers’ perceptions and experiences during the COVID-19 crisis: a qualitative study. Iran J Nurs Midwifery Res. (2021) 26:238–44. doi: 10.4103/ijnmr.IJNMR_285_20, PMID: 34277375 PMC8262538

[3] Labrague LJ De Los Santos JA. COVID-19 anxiety among front-line nurses: predictive role of organisational support, personal resilience and social support. J Nurs Manag. (2020) 28:1653–61. doi: 10.1111/jonm.13121, PMID: 32770780 PMC7436313

[4] Lee SH. Mental health impacts in health care workers during the COVID-19 pandemic. J Korean Neuropsychiatr Assoc. (2021) 60:19–22. doi: 10.4306/jknpa.2021.60.1.19

[5] Park JY Woo CH. The relationship among moral sensitivity, self-leadership, fatigue and compliance with standard precautions of intensive care nurses. J Digit Converg. (2020) 18:229–37.

[6] Wilson NM Norton A Young FP Collins DW. Airbone transmission of severe acute respiratory syndrome coronavirus-2 to healthcare workers: a narrative review. Anaesthesia. (2020) 75:1086–95. doi: 10.1111/anae.15093, PMID: 32311771 PMC7264768

[7] Choi KS Lee KH. Experience in responding to COVID-19 of nurse manager at a nursing hospital. J Humanit Soc Sci. (2020) 11:1307–22. doi: 10.22143/HSS21.11.5.94

[8] Jackson J Nowell L. The office of disaster management nurse managers’ experiences during COVID-19: a qualitative interview study using thematic analysis. J Nurs Manag. (2021) 29:2392–400. doi: 10.1111/jonm.13422, PMID: 34270140 PMC8420524

[9] Adams JG Walls RM. Supporting the health care workforce during the COVID-19 global epidemic. JAMA J Am Med Assoc. (2020) 323:1439–40. doi: 10.1001/jama.2020.3972 32163102

[10] Poortaghi S Shahmari M Ghobadi A. Exploring nursing managers’ perceptions of nursing workforce management during the outbreak of COVID-19: a content analysis study. BMC Nurs. (2021) 20:27. doi: 10.1186/s12912-021-00546-x 33514351 PMC7844784

[11] Jung YR Choi SY Seo MJ. The work experience of nurses in COVID-19 isolation wards. J Korean Clin Nurs Res. (2024) 30:304–15. doi: 10.22650/JKCNR.2024.30.3.304

[12] Woo KA Yun EK Choi JS Byun HM. Burnout among nurses in COVID-19 designated units compared with those in general units caring for both COVID-19 and non-COVID-19 patients. J Korean Acad Nurs Adm. (2023) 29:374–84. doi: 10.11111/jkana.2023.29.4.374

[13] Jeong E Jeong ML Kim YM. Influences of safety attitude for patient, and moral sensitivity in practice of standard precaution in small and medium hospital nurses. J Digit Converg. (2021) 19:453–61. doi: 10.14400/JDC.2021.19.9.453

[14] Lee NY Lee HJ. South Korean nurses' experiences with patient care at a COVID-19-designated hospital: growth after the frontline battle against an infectious disease pandemic. Int J Environ Res Public Health. (2020) 17:9015. doi: 10.3390/ijerph17239015, PMID: 33287343 PMC7729510

[15] Chung SJ Seong MH Park JY. Nurse’s experience in COVID-19 patient care. J Korean Acad Nurs Adm. (2022) 28:142–53. doi: 10.11111/jkana.2022.28.2.142

[16] Khattak SR Saeed I Rehman SU Fayaz M. Impact of fear of COVID-19 pandemic on the mental health of nurses in Pakistan. J Loss Trauma. (2021) 26:421–35. doi: 10.1080/15325024.2020.1814580

[17] Mo Y Deng L Zhang L Lang Q Liao C Wang N. Work stress among Chinese nurses to support Wuhan in fighting against COVID-19 epidemic. J Nurs Manag. (2020) 28:1002–9. doi: 10.1111/jonm.1301, PMID: 32255222 PMC7262235

[18] Vázquez-Calatayud M Regaira-Martínez E Rumeu-Casares C Paloma-Mora B Esain A Oroviogoicoechea C. Experiences of frontline nurse managers during the COVID-19: a qualitative study. J Nurs Manag. (2022) 30:79–89. doi: 10.1111/jonm.13488, PMID: 34592013 PMC8646738

[19] Moyo I Mgolozeli SE Risenga PR Mboweni SH Tshivhase L Mudau TS. Experiences of nurse managers during the COVID-19 outbreak in a selected district hospital in Limpopo Province, South Africa. Healthcare. (2021) 10:76. doi: 10.3390/healthcare10010076 35052240 PMC8775488

[20] Krueger RA Casey MA. Focus groups: a practical guide for applied research. 5th ed. Thousand Oaks (CA), USA: SAGE Publications (2015).

[21] Lincoln YS Guba EG. Naturalistic inquiry. Newbury Park (CA), USA: Sage Publications (1985), 9, 438–439.

[22] Hølge-Hazelton B Kjerholt M Rosted E Thestrup Hansen S Zacho Borre L McCormack B. Improving person-centred leadership: a qualitative study of ward managers' experiences during the COVID-19 crisis. Risk Manag Healthc Policy. (2021) 14:1401–11. doi: 10.2147/RMHP.S300648, PMID: 33854389 PMC8039537

[23] Leppäkoski T Mattila E Kaunonen M. Nursing managers' experiences of facing the COVID-19 pandemic in their work: a systematic review. Nurs Open. (2023) 10:4185–95. doi: 10.1002/nop2.1694, PMID: 36895077 PMC10277456

[24] Nicole B Michael AR. A systematic review of activities undertaken by the unregulated nursing assistant. J Adv Nurs. (2020) 76:1538–51. doi: 10.1111/jan.14354, PMID: 32190928

[25] Hosseinpour M Akbar RE Jahromi MF Badiyepeymaiejahromi Z. The effect of interventional program underpinned by health belief model on awareness, attitude, and performance of nurses in preventing nosocomial infections: a randomized controlled trial study. Invest Educ Enferm. (2023) 41:e10. doi: 10.17533/udea.iee.v41n3e10 PMC10990589 38589310

[26] Mansour SI Shosha GM. Experiences of first-line nurse managers during COVID-19: a Jordanian qualitative study. J Nurs Manag. (2022) 30:384–92. doi: 10.1111/jonm.13530, PMID: 34897860

[27] Jun J Ojemeni MM Kalamani R Tong J Crecelius ML. Relationship between nurse burnout, patient and organizational outcomes: systematic review. Int J Nurs Stud. (2021) 119:103933. doi: 10.1016/j.ijnurstu.2021.103933 33901940

[28] Hao P Wu L Liu Y. A survey on work status and competencies of clinical research nurses in China. J Res Nurs. (2022) 27:82–98. doi: 10.1177/17449871211067963, PMID: 35392202 PMC8980573

[29] Kwak SH Hyun SK. Exploring job stress, job satisfaction, and turnover intention of nurses in the comprehensive nursing service. J Convergence Cult Technol. (2019) 5:23–30. doi: 10.17703/JCCT.2019.5.2.23

