# Supplementary material for: Experiences of COVID-19 dedicated ward nurse managers in South Korea: a qualitative study
Source: Front Public Health. 2025 Oct 9;13:1675436. doi: 10.3389/fpubh.2025.1675436 (PMC12546330; doi:10.3389/fpubh.2025.1675436)
Supplement: Supplementary file 1 [file Presentation_1.pdf]

Appendix. Interview Questionnaire

A. General Characteristics

The following is about your general information. Please mark the appropriate box.

1. How old are you? ( ) years old

2. What is your gender? ① Male ② Female

3. What is your highest level of education?

① Associate degree ② Bachelor's degree ③ Master's degree or higher

4. What is your current hospital? ① Higher-level general hospital ② General hospital

5. How long have you worked as a total nursing manager? ( ) years ( ) months

6. How long have you worked in a COVID-19 ward? ( ) years ( ) months

B. Interview Questions

Step	Questions
Start	"Thank you for taking the time out of your busy schedule. Before we begin sharing our experiences, I would like to take a moment to introduce ourselves. Could you briefly introduce yourselves?"
Introduction	"What was your experience before managing a COVID-19 ward?" "When and how did you start working as a nurse manager on a COVID-19 ward?" "How has your experience managing the ward changed now compared to before you took on the role of COVID-19 ward manager?"
Transition	"If anything has changed, what specifically has changed?"
Key	"Can you tell us about your experience as a dedicated ward nurse manager during the COVID-19 pandemic?" "What challenges did you face while

	managing the COVID-19 ward? Why?" "How did you cope with these challenges?" - What was your role as a nurse manager in the face of these challenges? - What strategies did you use with your department members to manage the crisis? "What resources were needed for efficient dedicated ward management?" - What challenges did the hospital face in terms of resource requirements (human, material, environmental) related to COVID-19 response? "Based on your experience managing the dedicated ward, what do you think are the key aspects of crisis management in this regard?" "What did everything you experienced as a COVID-19 dedicated ward nurse manager mean to you?" - What are the key lessons you have learned during this period?
Closing	"Before we end the interview, is there anything else you would like to add? Do you have any questions regarding this matter?"
